# Calibur: a tool for clustering large numbers of protein decoys

**DOI:** 10.1186/1471-2105-11-25

**Published:** 2010-01-13

**Authors:** Shuai Cheng Li, Yen Kaow Ng

**Affiliations:** 1 David R. Cheriton School of Computer Science, University of Waterloo, Waterloo ON N2L 3G1 Canada; 2 Department of Computer and Information Sciences, Tokyo University of Agriculture and Technology, 2-24-16 Naka-cho, Koganei, Tokyo 184-8588, Japan

## Abstract

**Background:**

Ab initio protein structure prediction methods generate numerous structural candidates, which are referred to as decoys. The decoy with the most number of neighbors of up to a threshold distance is typically identified as the most representative decoy. However, the clustering of decoys needed for this criterion involves computations with runtimes that are at best quadratic in the number of decoys. As a result currently there is no tool that is designed to exactly cluster very large numbers of decoys, thus creating a bottleneck in the analysis.

**Results:**

Using three strategies aimed at enhancing performance (proximate decoys organization, preliminary screening via lower and upper bounds, outliers filtering) we designed and implemented a software tool for clustering decoys called Calibur. We show empirical results indicating the effectiveness of each of the strategies employed. The strategies are further fine-tuned according to their effectiveness.

Calibur demonstrated the ability to scale well with respect to increases in the number of decoys. For a sample size of approximately 30 thousand decoys, Calibur completed the analysis in one third of the time required when the strategies are not used.

For practical use Calibur is able to automatically discover from the input decoys a suitable threshold distance for clustering. Several methods for this discovery are implemented in Calibur, where by default a very fast one is used. Using the default method Calibur reported relatively good decoys in our tests.

**Conclusions:**

Calibur's ability to handle very large protein decoy sets makes it a useful tool for clustering decoys in ab initio protein structure prediction. As the number of decoys generated in these methods increases, we believe Calibur will come in important for progress in the field.

## Background

In ab initio protein structure predictions [1-5], it is often the case that a large set of candidates (called decoys) is generated, and one is required to select a single (or a small selection of) best candidate(s) from the set. One criterion for this selection is to choose decoys with more neighbors over decoys with fewer neighbors. The use of this criterion is well justified [6,7], and there are a few tools which incorporate this strategy [1,8,9]. In the popular protein structure prediction systems I-TASSER [2,8] and ROSETTA [1], decoys are selected using the following procedure: Starting with the set of generated decoys, a threshold *d *is first decided. Then, from the set, the decoy with the most neighboring decoys within RMSD *d *from it is found, and is reported as the highest ranking decoy. (Ties are broken arbitrarily.) This decoy and all of its neighbors (the first cluster) are then removed from the set, after which the decoy with the most neighbors within RMSD *d *is again found. This decoy is then reported as the second highest ranking decoy, and together with all its neighbors (the second cluster) are removed from the set. Similarly the third highest ranking decoy is then found, and so on.

Current implementations of this procedure evaluate pairwise RMSD (or approximate values) of the decoys, resulting in runtimes which are at best quadratic in the number of decoys. As the number of decoys grows to the tens of thousands, this method becomes infeasible, necessitating the development of faster methods. In this paper we propose three strategies to speed up the procedure, with no compromise on the clustering performed. That is, the resultant method produces exactly the same clusters that are produced based on pairwise comparison, but only faster. In the first strategy we create auxiliary groups of proximate decoys. This allows us to, through the use of triangular inequality, deduce if a group of decoys is (or is not) within the threshold distance from a given decoy. Our second strategy is to use efficiently computable lower and upper bounds of the RMSD to preliminary screen out unlikely candidates. Thirdly, outlier decoys can be detected and removed prior to the clustering. These strategies are implemented in an open-source tool called Calibur.

## Implementation

Coordinates of the C_*α *_atoms on the protein fold backbone are used to represent the main structure of a protein. As distance measure between two protein structures we use the backbone C_*α*_-carbon root mean squared deviation (C_*α *_RMSD). Each C_*α *_atom corresponds to a point in 3D space. For two protein structures *S*_1 _= (*s*_1,1_, *s*_1,2_, ..., *s*_1, *n*_) and *S*_2 _= (*s*_2,1_, *s*_2,2_, ..., *s*_2, *n*_), each *s*_*i*, *j*_, 1 ≤ *i *≤ 2, 1 ≤ *j *≤ *n*, is a 3D point indicating a C_*α *_atom in the backbone. The C_*α *_RMSD of *S*_1 _and *S*_2 _is defined as:

where ℛ is the set of all rotations and  the set of all translations.

### Strategy 1: Auxiliary grouping of decoys

**Figure 1:**
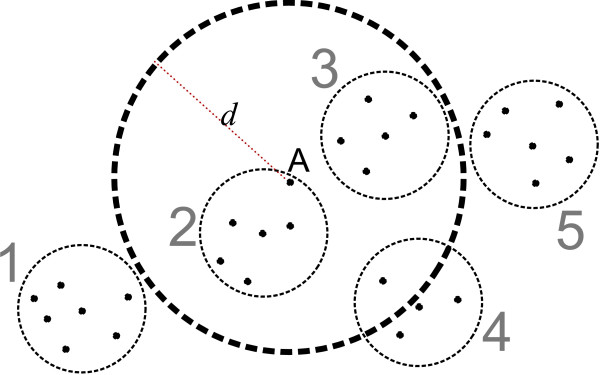
**Auxiliary grouping**. Using auxiliary grouping of decoys.

To avoid pairwise C_*α *_RMSD computation, proximate decoys can be considered collectively, in deciding whether they are within C_*α *_RMSD *d *from a decoy. We illustrate this idea in Figure 1, where the input decoys are collected into five groups. When finding all the decoys that are within C_*α *_RMSD *d *from the decoy *A *(which is itself in group 2), one can first consider each of the five groups as a whole. In this case, all the decoys in the groups 2 and 3 are within C_*α *_RMSD *d *from *A*, while all the decoys in the groups 1 and 5 are further than *d *from *A*. No such conclusion can be collectively made about the decoys in group 4. This strategy is made possible by the fact that we can decide if an entire collection of decoys is within C_*α *_RMSD *d *from a decoy *A *by comparing *A *to a representative decoy *C *for the collection. That is, if *A *is within a certain distance from *C*, then we conclude that the entire group is within *d *from *X*. Similarly, no decoy in the group is within *d *from *A *if *A *is further than some distance from *C*. How this can be done is as follows.

We want a grouping such that each decoy belongs to exactly one group, and is at most C_*α *_RMSD *r *from the group's *center *(i.e. the representative decoy). This is done as follows: First a distance *r *less than *d *is decided, and an arbitrary decoy is set as a center. (Let *r *=  for Case 1 below.) Repeatedly we take an ungrouped decoy, and try to find from all current centers for one which it is within distance *r *from. If and when any such center *C *is found the decoy is grouped with *C *and its distance to *C *is recorded. Otherwise the decoy is declared as a new center.

To locate the decoys in a group that are within distance *d *from a decoy *A*, one can consider the following five cases (denote by *C *the group's center and *X *an arbitrary decoy in the group):

Case 1: *A *is in the group of *C *(including when *A *is the group's center), given that *r *= .

Case 2: C_*α *_RMSD(*A, C*) + *r *≤ *d*.

Case 3: C_*α *_RMSD(*A, C*) > *d *+ *r*.

Case 4: C_*α *_RMSD(*A, C*) + C_*α *_RMSD(*C, X*) ≤ *d*

Case 5: |C_*α *_RMSD(*A, C*) - C_*α *_RMSD(*C, X*)| > *d*

**Figure 2:**
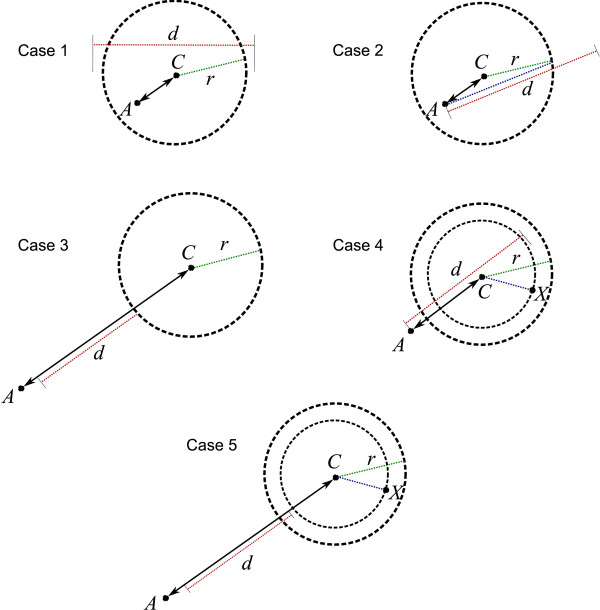
**Deciding if decoys in a group is within *d *from *A***. Deciding if decoys' distances are within the given threshold without exhaustive C_*α *_RMSD computation

These cases are depicted in Figure 2. Since C_*α *_RMSD is a metric [10], triangular inequality applies. Hence in Cases 1 and 2, all the decoys grouped with *C *must be within distance *d *from *A*. In Case 3 the converse is true.

In Cases 4 and 5, we take advantage of the already computed distance from the group's center to each member of the group. Again, triangular inequality implies that in Case 4, the decoy *X *is within distance *d *from *A*, while in Case 5 the converse is true. The C_*α *_RMSD between *X *and *A *is computed if and only if none of the cases applies.

### Strategy 2: Lower and upper bounds to C_*α *_RMSD

Given any two decoys *X *and *A*, an efficiently computable lower bound of C_*α *_RMSD(*X, A*) can be used to detect if C_*α *_RMSD(*X, A*) is larger than a given threshold *d*. Likewise, an upper bound can be used to detect the case where C_*α *_RMSD(*X, A*) is smaller than *d*. Our strategy is to use multiple such efficiently computable bounds as preliminary checks to reduce the much more expensive C_*α *_RMSD computations. We will first propose a few of such upper and lower bounds, and then demonstrate how they are applied. First consider any three decoys, *O*, *X *and *Y*. By triangular inequality,

Hence we can efficiently compute an upper and a lower bound to C_*α *_RMSD(X, Y), through an arbitrarily chosen *reference decoy O *and pre-computed C_*α *_RMSD(*X, O*) values for each decoy *X*. In practice, one can use *n *reference decoys *O*_1_, *O*_2_, ..., *O*_*n *_to obtain *n *upper bounds and *n *lower bounds.

The Euclidean distance between two decoys, after they are re-orientated to minimize their C_*α *_RMSDs to a fixed arbitrary decoy, yields another upper bound to their C_*α *_RMSD [9]. This upper bound distance is referred to as rRMSD.

Another lower bound can be obtained as follows. Denote the centroid of a protein structure *S*_*x *_as *c*_*x*_. The *signature Sig*_*x *_for a protein structure *S*_*x *_= (*s*_*x*, 1_, *s*_*x*, 2_, ..., *s*_*x*, *n*_) is defined as:(1)

where *v*_*x*, *i *_= ||*s*_*x*, *i *_- *c*_*x*_||, 1 ≤ *i *≤ *n*. Define the distance between two signatures *Sig*_1 _and *Sig*_2_, called *signature distance*, as:(2)

The signature distance of two protein structures is a lower bound of their C_*α *_RMSD, that is:

**Lemma 1**. *C_*α *_RMSD*(*S*_1_, *S*_2_) ≥ *dist*(*Sig*_1_, *Sig*_2_)

*Proof*. Let *R *and *T *be the optimal rotation and translation found in computing the C_*α *_RMSD of two structures *S*_1 _and *S*_2_. Let *r*_*k *_= ||*Rs*_1, *k *_- *s*_2, *k*_- *T*||^2^, *u*_1, *k *_= ⟨*s*_1, *k*_, *c*_1_⟩ and *u*_2, *k *_= ⟨*s*_2, *k*_, *c*_2_⟩, 1 ≤ *k *≤ *n*. *u*_1, *k *_and *u*_2, *k *_are line segments with lengths *v*_1, *k *_and *v*_2, *k *_respectively.

It is known that the superposition in computing the C_*α *_RMSD of any two structures results in the centroids of the structures to coincide [11].

Let *θ *be the angle between *u*_1, *k *_and *u*_2, *k*_. By trigonometry, . Hence, C_*α *_RMSD (*S*_1_, *S*_2_) = .

To decide if a decoy *X *is within C_*α *_RMSD *d *of a decoy *A*, we first compute the bounds and examine the following.

• If any of the upper bounds of C_*α *_RMSD(*X, A*) is smaller than or equal to *d*. If so, clearly C_*α *_RMSD(*X, A*) ≤ *d*.

• If any of the lower bounds of C_*α *_RMSD(*X, A*) is larger than *d*. If so, clearly C_*α *_RMSD(*X, A*) > *d*.

We compute C_*α *_RMSD(*X, A*) if and only if these two checks fail.

The upper and lower bounds can also be applied to the conditions in Case 2 and Case 3 of Strategy 1, as follows.

• In Case 2, if any of the upper bounds of C_*α *_RMSD(*A, C*) is smaller than *d *- *r*, then the condition C_*α *_RMSD(*A, C*) + *r *≤ *d *holds.

• In Case 3, if any of the lower bounds of C_*α *_RMSD(*A, C*) is larger than *d *+ *r*, then the condition C_*α *_RMSD(*A, C*) > *d *+ *r *holds.

We compute C_*α *_RMSD(*A, C*) for Case 2 and Case 3 if and only if these two checks fail.

### Strategy 3: Filtering outlier decoys

Another possible enhancement to performance is to discard decoys with low similarity to other decoys in the set, prior to the clustering. Here we propose an efficient technique to quickly identify such decoys. Our aim is to retain all of the high ranking decoys, and the decoys which are within distance *d *from them. We identify these as "good" decoys. Assume that every high ranking decoy is within distance *d *from 10% of all the decoys. For a random sample of *n *decoys, the probability for a good decoy to be within a distance 2*d *from at least one of the sampled decoys is 1 - 0.9^*n*^, which is > 0.9999 when *n *= 100. Hence decoys that are not within 2*d *from any one of 100 randomly sampled decoys are likely not good, and are removed from the set.

### Overall program design

We designed a program based on the three strategies. On a given set *S *of *n *input decoys, the program does the following:

Step 1: Discover a suitable threshold distance *d *for clustering *S*.

Step 2: Filter outlier decoys using 100 randomly selected decoys, as in Strategy 3.

Step 3: Create auxiliary groups out of the decoys as required by Strategy 1. Compute the signature (*Sig*_*x*_), and the distances (C_*α *_RMSD(*X, O*) for each decoy *X *and reference decoy *O*; rRMSD(*X, Y*) for each decoy *X*, *Y*) as required by Strategy 2.

Step 4: Find for each decoy *A *a neighbor set *N*_*A *_which contains all the decoys in *S *within distance *d *from *A *(*A *inclusive), using Strategy 1 with the preliminary checks of Strategy 2. This is done in a straight-forward fashion as follows.

   1. Set *N*_*A *_to an empty set.

   2. For each auxiliary group of decoys *G *(*C *denote the center of *G*),

      (a) If *A *is in *G*, add all decoys in *G *into *N*_*A *_and go for the next auxiliary group.

      (b) Examine if C_*α *_RMSD(*A, C*) + *r *≤ *d *using each of the upperbounds of C_*α *_RMSD(*A, C*).

         If true, add all decoys in *G *into *N*_*A*_. Go for the next auxiliary group.

      (c) Examine if C_*α *_RMSD(*A, C*) > *d *+ *r *using each of the lowerbounds of C_*α *_RMSD(*A, C*).

         If true, skip *G*. Go for the next auxiliary group.

      (d) Compute C_*α *_RMSD(*A, C*).

      (e) Examine if C_*α *_RMSD(*A, C*) + *r *≤ *d*.

         If true, add all decoys in *G *into *N*_*A*_. Go for the next auxiliary group.

      (f) Examine if C_*α *_RMSD(*A, C*) > *d *+ *r*. If true, skip *G*. Go for the next auxiliary group.

      (g) For each decoy *X *in *G*,

            i. Examine if C_*α *_RMSD(*A, C*) + C_*α *_RMSD(*C, X*) ≤ *d*.

               If true, add *X *into *N*_*A*_. Go for the next decoy in *G*.

            ii. Examine if |C_*α *_RMSD(*A, C*) - C_*α *_RMSD(*C, X*)| > *d*.

               If true, skip *X*. Go for the next decoy in *G*.

            iii. Examine if C_*α *_RMSD(*A, X*) ≤ *d *using each of the upperbounds of C_*α *_RMSD(*A, X*).

               If true, add *X *into *N*_*A*_. Go for the next decoy in *G*.

            iv. Examine if C_*α *_RMSD(*A, X*) > *d *using each of the lowerbounds of C_*α *_RMSD(*A, X*).

               If true, skip *X*. Go for the next decoy in *G*.

            v. Compute C_*α *_RMSD(*A, X*).

            vi. If C_*α *_RMSD(*A, X*) ≤ *d*, add *X *into *N*_*A*_.

   3. Output *N*_*A*_.

Step 5: Start with an empty sequence *Output*. Repeatedly find *A *∈ *S *with the largest *N*_*A*_, appending *A *to *Output *while removing *N*_*A *_from *S *and all the neighbor sets.

Step 6: Output the decoys in *Output*. (For brevity the program is set to output only the first 3 decoys.)

The threshold selection in Step 1 is addressed in the next sub-section.

Steps 2 and 3 are performed straightforwardly.

Step 5 is performed by repeating the following until *S *is empty: Find the decoy *X *∈ *S *with the largest *N*_*X *_(breaking ties arbitrarily) and append the decoy to *Output*. Then, remove *N*_*X *_from *S *and for each *Y *∈ *N*_*X*_, remove *Y *from *N*_*Z *_for each *Z *∈ *N*_*Y*_.

#### Selection of a suitable threshold

We consider two decoys to be significantly related if and only if their C_*α *_RMSD is *relatively small *among all pairwise C_*α *_RMSDs of the decoys. Hence our strategy to threshold finding is to identify a value *d *such that only *x *percent of pairwise C_*α *_RMSD distances are below *d*, for some suitable *x*. Given *x*, a straightforward way to determine such a *d *exactly is to compute all *n *× *n *C_*α *_RMSDs and find the (0.01*xn*^2^)-th shortest distance. Alternatively, a reasonable approximation to the *x*-percentile value can be obtained efficiently using only a relatively small random sample of the decoys. In our tests, around 10 samplings of 100 decoys each sufficed to determine this value quickly and consistently in general. Our program uses this method by default, with *x *set to min{100*n*^-1/4^, 10}. The expression 100*n*^-1/4 ^is heuristic. It's aim is to reduce the percentile when more decoys are available, in order to speed up the clustering (e.g., *x *= 10 when *n *= 10000, *x *= 5 when *n *= 160000).

A similar strategy would be to use the most frequently occurring C_*α *_RMSD among decoys, *f *say, as a reference to decide a threshold distance *d*. (If the pairwise distances are distributed normally, *f *would correspond to the 50th percentile.) As a selectable option the program includes a simple method based on this, in which we let *d *= *cf *+ *b*, where *c *is set to  and *b *is set to the minimum pairwise distance discovered through random sampling.

#### Memory usage

In Steps 1-3 and 5, the memory required is linear in *n*. For Step 4, in the theoretical worst case, |*N*_*X*_| = *n *for each *X*, resulting in *O*(*n*^2^) memory usage. However, such a scenario is unlikely to occur in the program's intended use. In practical use, |*N*_*X*_| is seldom above 0.2*n*, and small for most *X*. Note that in the case that the number of neighbor sets of a given size falls off geometrically with the size, the memory required to store all neighbor sets would in fact be linear in *n*. In our tests, the actual growth in memory usage is closer to *O*(*n*) than *O*(*n*^2^).

If one is interested in only the highest ranked decoy from the clustering, it is unnecessary to construct the neighbor sets, since the sizes of the neighbor sets suffice to determine such a decoy. In this case, the total memory usage would be linear in *n*. We include this mode of operation as an option.

## Results

Our C++ implementation of the program is called Calibur. Calibur accepts as input a list of names of PDB files (each for a decoy) and an optional threshold *d*. No pre-processing is required of the PDB files. If no threshold is given, Calibur automatically finds a suitable threshold for the input decoys, as discussed. The method which Calibur uses for threshold discovery can be altered through commandline arguments. A list of all the implemented methods is shown when Calibur is called without any input arguments.

### Effectiveness of strategies

The effectiveness of each of the strategies was evaluated with decoys predicted by FALCON (reported in Alipanali *et al.*: A protocol for automated NMR protein structure determination, submitted for publication) on the proteins TM1112 from the Arrowsmith Lab at University of Toronto (herein the set is referred to as TM1112) and SH3 from Donaldson's Lab at York University (herein referred to as CASKIN). Each of these two sets contains 9999 decoys.

#### Auxiliary grouping, lower and upper bounds

**Figure 3:**
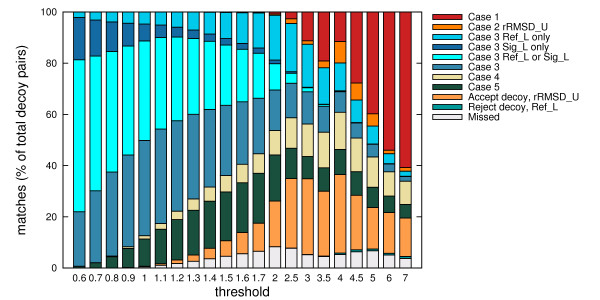
**Contribution of Strategies 1 and 2 on TM1112**. The number of C_*α *_RMSD computations avoided (percentage over 9999 × 9998 computations) due to each of the cases considered, at different threshold values. For Case 2, the upper bounds are used for condition evaluation prior to the actual C_*α *_RMSD. The contribution from the upper bounds via the reference decoys can be completely accounted for by rRMSD, while C_*α *_RMSD evaluations contributed insignificantly. Only the contribution from rRMSD (label "Case 2 rRMSD_U") is shown. For Case 3, the lower bounds are used for condition evaluation prior to the actual C_*α *_RMSD. While the contributions from both kinds of lower bounds overlapped (label "Case 3_Ref_L or Sig_L"), there were contributions entirely due to evaluations using the signature (label "Case 3 Sig_L only") as well as the reference decoys (label "Case 3 Ref_L only"). The contribution from evaluating the actual C_*α *_RMSD was highly significant as well (label "Case 3"). In evaluating individual decoys, the upper bound obtained from rRMSD was highly effective at high thresholds (label "Accept decoy, rRMSD_U"). The lower bounds from the reference decoys demonstrated noticeable effects (label "Reject decoy, Ref_L"). Other contributions were insignificant.

**Figure 4:**
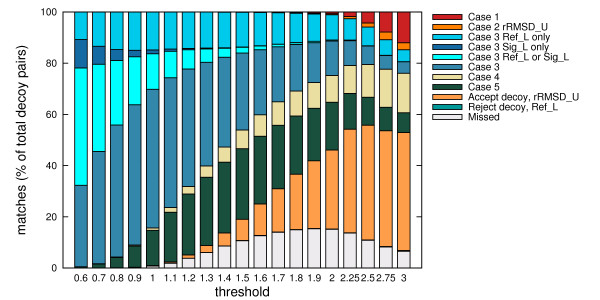
**Contribution of Strategies 1 and 2 on CASKIN**. Same as Figure 3, but on the CASKIN data set.

Each of the different cases contributed in reducing the runtime, although the amounts differed at different thresholds (see Figures 3 and 4). At low thresholds, the chances of decoys being further than the threshold distance are high. Hence evaluations via Case 3 and the lower bounds are more effective. For a similar reason, the effects of evaluations through Case 1 and the upper bounds become elevated at larger thresholds.

In Calibur, the order in which evaluations are performed, as well as the range of thresholds to use for the evaluations has been optimized based on these observations.

#### Filtering

**Figure 5:**
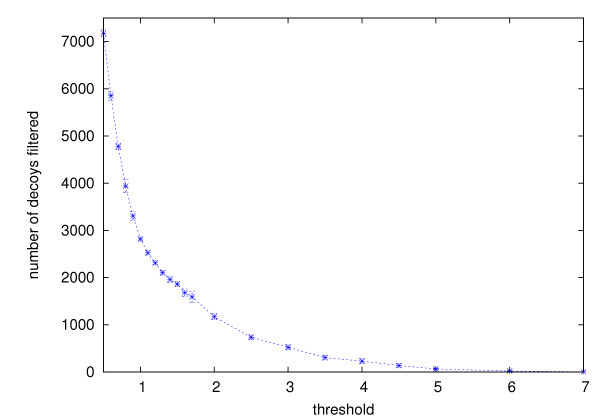
**Number of decoys filtered from TM1112**. The number of decoys filtered from the set TM1112 using 100 randomly selected decoys at different thresholds. Each value is an average of 10 numbers from 10 different trials using the same threshold. Error bars show the standard deviations.

**Figure 6:**
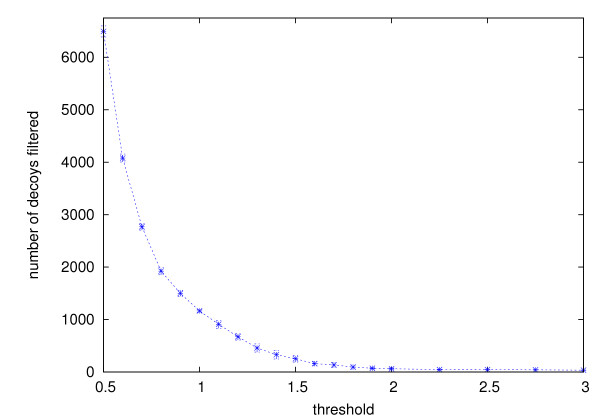
**Number of decoys filtered from CASKIN**. Same as Figure 5, but on the CASKIN data set.

On the data sets TM1112 and CASKIN, filtering did not affect the clusters formed by the highest ranking decoys. Their rankings remained the same. This is true even in the cases where more than 70% of decoys had been filtered prior to the clustering. Figure 5 and 6 show, for TM1112 and CASKIN respectively, the number of decoys filtered (out of the total of 9999 decoys) at various threshold values.

#### Strategies' effects on Calibur's performance

To evaluate the strategies' effects on Calibur at various thresholds, the runtimes when the strategies are used ("Calibur") and when they are not used ("*pairwise*") were compared. For reference purposes, the runtime for ROSETTA's pairwise evaluation based clustering program ("cluster_info_silent") is also shown. ROSETTA is currently the most popular system for protein structure prediction. All the tests are run on a 3 GHz Intel Core 2 Duo PC with 2.98 G RAM running CentOS 5.3. All three tools are compiled using GCC 4.1.2 with optimization -O. The same codes is used for computing C_*α *_RMSD.

**Figure 7:**
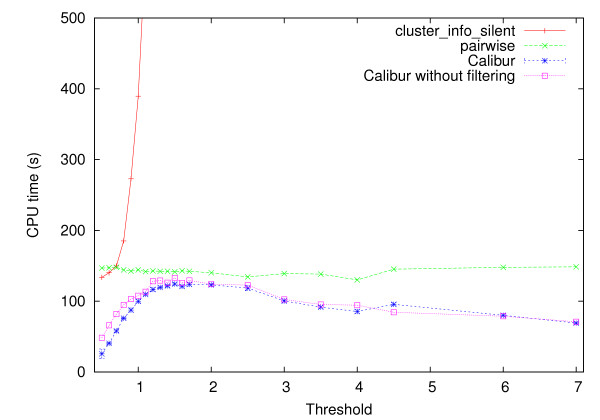
**Runtime of cluster_info_silent, pairwise and Calibur on TM1112**. The CPU times used to obtain cluster at different thresholds on the TM1112 data set of 9999 decoys, by (1) cluster_info_silent (label "cluster_info_silent"). (2) Calibur without using any of the strategies (label "*pairwise*"), (3) Calibur (label "Calibur") (To account for variations caused by the filtering each point is an average of 10 trials), (4) Calibur with the filtering mechanism disabled (label "Calibur without filtering").

All the tools were given input such that the output would be exactly the same. Hence we compare only their runtimes. For *pairwise *and cluster_info_silent, the CPU time is taken to be the total time needed for neighbors finding and the recursive search for largest clusters. For Calibur, the CPU time is the sum of the times taken for signature computation, decoys re-orientation, filtering, auxiliary grouping, neighbors finding, and the recursive search for the largest clusters. Figure 7 shows the results on the data set TM1112. The largest sizes of the clusters at the thresholds 1, 2, 3, 4, 5, 6, 7 are respectively 1796, 6017, 7744, 8186, 8671, 9120, 9368.

### Calibur's performance on a large data set

Calibur's performance in handling large numbers of decoys was evaluated using a set of 29770 decoys for the TM1112 protein generated by FALCON. For each threshold in 0.5, 1, 1.5, 2, 3, 4, 5, we performed 10 trial runs over a UNIX cluster. (More precisely, an HP XC cluster with 378 nodes, each with 8× Xeon 3.0 GHz CPUs and 16 GB memory, running RHEL 5.1.) All the runs resulted in the same decoy clusters. Table 1 shows the average CPU times (in sec).

**Table 1:** CPU times of Calibur on large data set.

Threshold	0.5 (27)	1.0 (1966)	1.5 (5531)	2.0 (8560)	3.0 (14397)	4.0 (17915)	5.0 (19905)
Calibur	74 ± 7	506 ± 14	1047 ± 27	1482 ± 42	2369 ± 154	3109 ± 266	3616 ± 290
no filtering	225 ± 11	717 ± 22	1250 ± 35	1629 ± 42	2495 ± 180	3166 ± 233	3501 ± 272
*Pairwise*	2628 ± 72	2624 ± 69	2651 ± 66	2741 ± 83	3293 ± 205	4014 ± 130	4425 ± 324

Numbers in brackets are the sizes of the largest clusters at the corresponding threshold.

CPU times of (1) Calibur, (2) Calibur with the filtering mechanism disabled ("no filtering"), and (3) *pairwise*, on a set of 29770 decoys for the TM1112 protein. Shown with ± standard deviation.

In practice the largest clusters typically contain around 10% of the decoys. In the present case, the largest clusters found at 1.5 threshold distance already contain more than 18% of all the decoys. At this point, the corresponding CPU time required by Calibur is about one third of the time required when the strategies are not used.

As a further reference on Calibur's performance in high load use, Calibur completed in around 15000 seconds CPU time under its default settings in our recent tests using 100,000 decoys.

### Evaluation of Calibur's output decoys

To evaluate the decoys produced by Calibur, we compared them to that obtained using SPICKER [8], the clustering tool used in the leading ab initio protein structure prediction system I-TASSER [2]. We used the decoy sets, natives and SPICKER's results published on I-TASSER's website [12], downloaded on the 24th of July, 2009. The data consists of decoys for 56 targets. The number of decoys for each target is shown in Table 2.

**Table 2:** Sizes of the decoy set for each target.

Target	#Decoys	Target	#Decoys	Target	#Decoys	Target	#Decoys
1abv_	12500	1dtjA_	20000	1mkyA3	6119	1shfA	20000
1af7_ _	12499	1egxA	20000	1mla_2	12500	1sro_	20000
1ah9_	27498	1fadA	12599	1mn8A	12500	1ten_	20000
1aoy_	32000	1fo5A	20000	1n0uA4	12499	1tfi_	32000
1b4bA	12500	1g1cA	19997	1ne3A	12500	1thx_	32000
1b72A	12499	1gjxA	12500	1no5A	12500	1tif_	12500
1bm8_	20000	1gnuA	17533	1npsA	20000	1tig_	12500
1bq9A	20000	1gpt_	32000	1o2fB_	12500	1vcc_	20000
1cewI	19830	1gyvA	11508	1of9A	20000	256bA	20000
1cqkA	19999	1hbkA	20000	1ogwA_	19998	2a0b_	32000
1csp_	12500	1itpA	12500	1orgA	20000	2cr7A	12500
1cy5A	32000	1jnuA	20000	1pgx_	20000	2f3nA	19999
1dcjA_	20000	1kjs_	20000	1r69_	20000	2pcy_	20000
1di2A_	20000	1kviA	20000	1sfp_	19985	2reb_2	12500

The number of decoys for each target.

In order to compare Calibur with SPICKER in terms of both output and speed we ran Calibur under the same conditions as SPICKER. Both programs were compiled with optimization -O3 and were made to cluster exactly the same set of decoys. Filtering was disabled in Calibur. We noticed a limit on the number of decoys that SPICKER handles. When the number of decoys is larger than 13000, SPICKER samples only 13000 decoys for clustering. To test Calibur with the same set of decoys that SPICKER clusters, we obtained 13000 decoys from each decoy set that is larger than 13000 (using the same procedure as in SPICKER's source codes) and tested Calibur with these decoys.

When decoys are sampled, they may not be sufficiently representative and the quality of the best decoy obtained may be compromised. To investigate this effect, we randomly sampled 1000, 2500, 4000, 5500, 7000, 8500, 10000, 11500 decoys from each of the original sets and ran SPICKER and Calibur with these sampled sets. Since only 6119 decoys are available for 1mkyA3, the full set was used as the sampled set at sizes above 5500.

**Figure 8:**
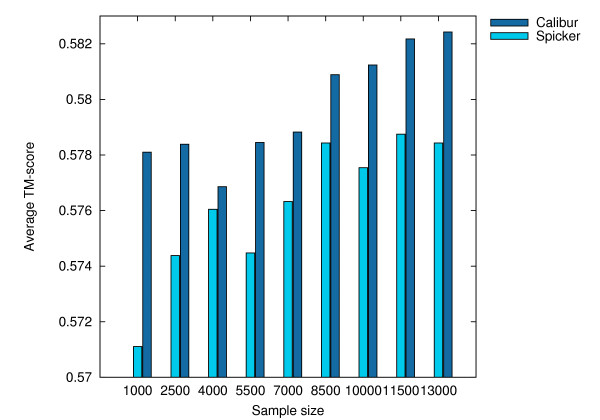
**Average TM-scores for the sample sets at different sizes**. Average TM-score for the best decoys obtained by SPICKER and Calibur respectively on the sampled sets of sizes 1000, 2500, 4000, 5500, 7000, 8500, 10000, 11500, 13000.

**Figure 9:**
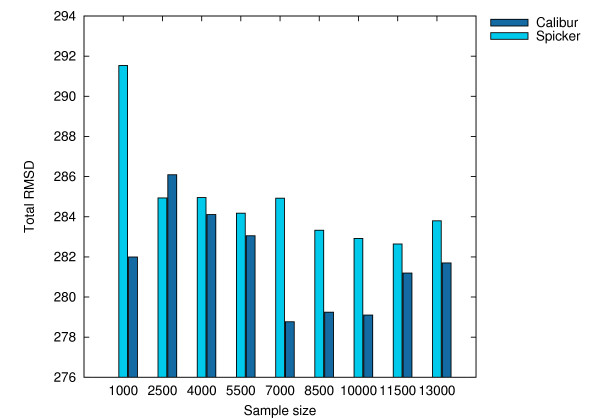
**Total C_α _RMSDs for the sample sets at different sizes**. Total C_*α *_RMSD for the best decoys obtained by SPICKER and Calibur respectively on the sampled sets of sizes 1000, 2500, 4000, 5500, 7000, 8500, 10000, 11500, 13000.

**Figure 10:**
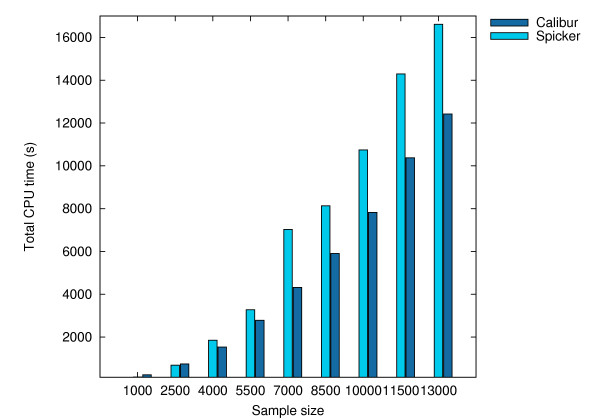
**Total CPU times for the sample sets at different sizes**. Total CPU time (sec) used by SPICKER and Calibur respectively on the sampled sets of sizes 1000, 2500, 4000, 5500, 7000, 8500, 10000, 11500, 13000.

All the tests were performed on the same UNIX cluster as in the previous section. Calibur used its default method for automatic threshold distance discovery. Table 3 shows, at different sample sizes, the average TM-scores and total C_*α *_RMSDs (to native) for the best decoys reported by both tools, as well as the total CPU times used, as reported by the UNIX servers. These results are shown as histograms in Figures 8, 9 and 10. Detailed results are given in Additional File 1. The average TM-score and total C_*α *_RMSD reported by both tools are similar, showing the decoys reported by both tools to be comparable. The CPU times required by Calibur are significantly less than SPICKER for sample sizes above 2500, even with PDB files processing time included. There is an observable trend of increase in average TM-score as well as decrease in total C_*α *_RMSD, with an increase in sample size.

**Table 3:** Total scores and CPU times.

Sample size	Average TM-score		Total C_*α *_RMSD		Total CPU Time (s)	
			
	SPICKER	Calibur	SPICKER	Calibur	SPICKER	Calibur
1000	0.571107	0.578102	291.534	281.994	136.05	237.25
2500	0.574379	0.578386	284.937	286.09	687.45	746.84
4000	0.576045	0.576859	284.953	284.108	1851.34	1533.81
5500	0.574475	0.57845	284.18	283.055	3276.36	2781.55
7000	0.576323	0.578823	284.927	278.769	7029.09	4320.95
8500	0.57843	0.580889	283.325	279.248	8132.9	5906.61
10000	0.577543	0.581236	282.919	279.1	10745.5	7822.75
11500	0.578748	0.582175	282.644	281.192	14293.8	10374.2
13000	0.578432	0.582425	283.795	281.701	16608.8	12420.5

Average TM-score and total C_*α *_RMSD for the best decoys obtained, as well as the total CPU time used, on the sampled sets of sizes 1000, 2500, 4000, 5500, 7000, 8500, 10000, 11500, 13000. (SPICKER requires additional pre-processing of the input PDB files which are not added into the CPU times here.)

**Figure 11:**
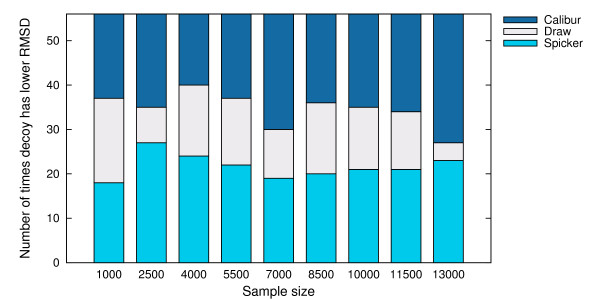
**Number of times decoy with better TM-score is reported by each method**. Number of times the decoy with better TM-score was reported by SPICKER and Calibur respectively, on the sampled sets of sizes 1000, 2500, 4000, 5500, 7000, 8500, 10000, 11500, 13000.

**Figure 12:**
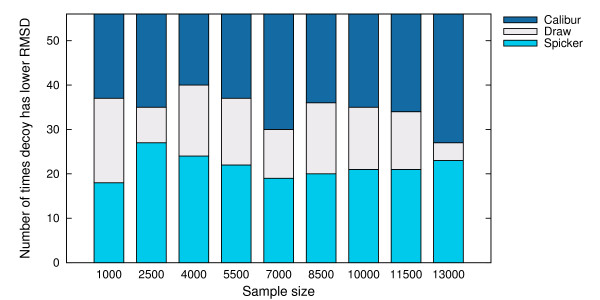
**Number of times decoy with better C_*α *_RMSD is reported by each method**. Number of times the decoy with better C_*α *_RMSD was reported by SPICKER and Calibur respectively, on the sampled sets of sizes 1000, 2500, 4000, 5500, 7000, 8500, 10000, 11500, 13000.

For each sample size, we counted the number of targets where the best decoy reported by SPICKER has a better TM-score than Calibur, as well as the number of targets where the best decoy reported by Calibur has a better TM-score. These numbers are shown in Figure 11. Figure 12 shows the numbers where instead of the TM-score, the C_*α *_RMSD is compared.

Further experiment with Calibur using more decoys than 13000 suggests that decoy quality may improve when a fuller set of decoys is used. We randomly sampled 16000, 19000, 22000, 25000, 28000 and 31000 decoys from each of the target sets and ran Calibur on these sampled sets. At each of the sample sizes, if the set contains less decoys than the sample size, then the entire set of decoys was used. The results are shown in Table 4 (details given in Additional File 2). The highest average TM-score and total C_*α *_RMSD is observed at sample size 28000. Rank sum test shows the average TM-scores obtained at the sample sizes above 13000 to be higher than those obtained at sample sizes less than or equal to 13000 (*p *≤ 0.1).

**Table 4:** Total scores and CPU times at larger sample sizes.

Max sample size	Average TM-score	Total C_*α *_RMSD	Total CPU Time (s)
16000	0.581143	283.749	28857.2
19000	0.582325	281.589	38928.9
22000	0.580996	279.887	45916
25000	0.581545	283.549	47083.1
28000	0.58272	278.243	50669.4
31000	0.581805	282.32	53582.2

Average TM-score and total C_*α *_RMSD for the best decoys obtained, as well as the total CPU time used, on the sampled sets of maximum sizes 16000, 19000, 22000, 25000, 28000 and 31000.

## Conclusion

Calibur is a carefully implemented tool, dedicated to the purpose of clustering very large numbers of decoys. As methods in ab initio protein structure prediction advances, the number of decoys to be analyzed is expected to increase, and the disability to cluster decoys efficiently will eventually pose a hindrance to the analyses of various problems and subproblems in the prediction of protein structures. It is our belief that Calibur, together with the methods it implements, will come in very useful when this situation arises. For this reason, we have decided to release the source codes of Calibur with an open license.

## Availability and requirements

• **Project name**: Calibur

• **Project homepage**: http://sourceforge.net/projects/calibur/

• **Operating System(s)**: Multiple platform (tested on Windows and Linux)

• **Programming Language**: C++.

• **Other requirements**: None.

• **License**: GNU General Public License

## Authors' contributions

All authors jointly developed the methods and wrote the article. They read and approved the final manuscript.

## Supplementary Material

Additional file 1 Details of experiments using sample sets of sizes 1000, 2500, 4000, 5500, 7000, 8500, 10000, 11500, 13000. Click here for file

Additional file 2 Details of experiments using sample sets of sizes 16000, 19000, 22000, 25000, 28000 and 31000. Click here for file
