# Supplementary material for: Calibur: a tool for clustering large numbers of protein decoys
Source: BMC Bioinformatics. 2010 Jan 13;11:25. doi: 10.1186/1471-2105-11-25 (PMC2881085; doi:10.1186/1471-2105-11-25)
Supplement: Additional file 1 — Details of experiments using sample sets of sizes 1000, 2500, 4000, 5500, 7000, 8500, 10000, 11500, 13000. [file 1471-2105-11-25-S1.pdf]

Sample size 1000

Target	TM-score		C_α RMSD		CPU Time	
	SPICKER	Calibur	SPICKER	Calibur	SPICKER	Calibur
1abv_	0.2962	0.2868	13.969	13.050	3.43	2.54
1af7__	0.4749	0.5320	4.479	3.508	2.87	1.93
1ah9_	0.4530	0.6848	5.143	2.763	3.10	2.29
1aoy_	0.6555	0.6555	4.773	4.773	3.62	2.19
1b4bA	0.4099	0.4850	6.578	5.565	3.20	2.40
1b72A	0.5945	0.6764	3.764	3.102	2.50	1.95
1bm8_	0.3499	0.3545	8.083	6.916	5.08	2.53
1bq9A	0.3777	0.3777	7.849	7.849	2.62	1.81
1cewI	0.1806	0.1737	16.241	16.052	6.19	3.07
1cqkA	0.8512	0.8301	1.756	1.841	6.11	2.94
1csp_	0.7187	0.7187	2.314	2.314	4.39	2.25
1cy5A	0.8798	0.8798	1.674	1.674	5.75	2.72
1dcjA_	0.3537	0.3719	11.868	12.051	3.83	2.30
1di2A_	0.7303	0.7677	2.699	2.696	3.61	2.30
1dtjA_	0.8113	0.7583	1.991	2.144	3.92	2.42
1egxA	0.7671	0.7671	2.635	2.635	6.71	3.19
1fadA	0.5800	0.5800	3.620	3.620	4.70	2.81
1fo5A	0.5353	0.5356	3.913	3.948	4.35	2.53
1g1cA	0.7812	0.7812	2.499	2.499	6.05	2.81
1gjxA	0.4396	0.3868	7.298	7.812	3.00	2.08
1gnuA	0.5472	0.5393	8.630	9.184	4.73	2.89
1gpt_	0.5043	0.5207	5.688	6.445	2.69	1.80
1gyvA	0.7583	0.7615	3.501	3.445	5.65	3.59
1hbkA	0.6662	0.6662	3.473	3.473	4.28	2.71
1itpA	0.3268	0.3222	11.699	11.174	2.86	1.89
1jnuA	0.7242	0.7536	2.830	2.615	5.22	3.05
1kjs_	0.3683	0.3683	8.479	8.479	3.95	2.36
1kviA	0.6841	0.6919	2.338	2.159	4.36	2.25
1mkyA3	0.4238	0.4238	5.028	5.028	4.16	2.64
1mla_2	0.6226	0.5557	3.137	3.258	3.60	2.27
1mn8A	0.3550	0.3015	7.179	7.809	3.71	2.24
1n0uA4	0.4661	0.4661	4.551	4.551	3.79	2.32
1ne3A	0.4022	0.5059	5.995	3.962	4.42	1.79
1no5A	0.3873	0.3888	10.839	10.771	4.24	2.77
1npsA	0.7686	0.7591	2.287	2.337	5.24	2.66
1o2fB_	0.3605	0.3424	8.676	9.279	3.79	1.99
1of9A	0.5416	0.5416	3.637	3.637	4.13	2.40
1ogwA_	0.6919	0.6919	2.720	2.720	3.84	2.23
1orgA	0.7546	0.7551	2.672	2.706	5.74	3.16
1pgx_	0.4997	0.4997	3.337	3.337	3.00	2.10
1r69_	0.7528	0.7528	2.035	2.035	3.31	2.11
1sfp_	0.7244	0.7437	5.250	5.169	5.62	3.09
1shfA	0.8411	0.8053	1.312	1.553	3.54	2.13
1sro_	0.6339	0.6246	3.566	3.779	4.60	2.31
1ten_	0.7892	0.8088	1.998	1.866	4.49	2.53
1tfi_	0.5158	0.5158	5.108	5.108	2.97	1.84
1thx_	0.7867	0.7867	2.407	2.407	5.42	2.97
1tif_	0.3299	0.3295	7.448	7.750	3.80	1.85
1tig_	0.4964	0.5278	9.579	4.108	3.57	2.25
1vcc_	0.3933	0.3933	8.113	8.113	3.60	2.07
256bA	0.7525	0.7389	3.506	3.519	5.38	2.94
2a0b_	0.7742	0.8051	2.748	2.470	6.80	3.21
2cr7A	0.4067	0.3715	4.890	8.330	4.87	1.82
2f3nA	0.7188	0.7188	1.960	1.960	3.54	2.17
2pcy_	0.6402	0.6594	4.658	4.691	4.60	2.79
2reb_2	0.3324	0.3328	7.114	5.955	2.71	1.80
	Avg. TM-score		Total C_α RMSD		Total CPU time	
	0.571107	0.578102	291.534	281.994	136.05	237.25

Sample size 2500

Target	TM-score		C_α RMSD		CPU Time	
	SPICKER	Calibur	SPICKER	Calibur	SPICKER	Calibur
1abv_	0.2719	0.2932	13.083	13.065	8.69	13.22
1af7__	0.4604	0.5140	4.899	4.296	7.51	9.88
1ah9_	0.5082	0.6591	4.701	2.853	9.51	11.84
1aoy_	0.6628	0.6628	4.818	4.818	13.35	13.59
1b4bA	0.4099	0.4705	6.578	5.758	9.09	14.23
1b72A	0.6631	0.6759	3.035	3.213	6.78	10.52
1bm8_	0.3617	0.3956	8.039	7.920	17.75	13.28
1bq9A	0.3608	0.3451	7.963	8.015	8.23	10.99
1cewI	0.1798	0.1746	16.219	16.112	17.92	19.21
1cqkA	0.8581	0.8328	1.708	1.844	18.89	14.44
1csp_	0.7199	0.7187	2.367	2.314	13.41	12.12
1cy5A	0.8684	0.8684	1.587	1.587	17.35	13.13
1dcjA_	0.3542	0.3522	10.798	11.968	9.11	9.99
1di2A_	0.7747	0.7665	2.408	2.500	12.32	11.68
1dtjA_	0.7821	0.7960	2.180	2.115	14.79	12.94
1egxA	0.7737	0.7644	2.551	2.651	20.88	15.66
1fadA	0.5746	0.5917	3.669	3.536	21.63	14.72
1fo5A	0.5318	0.5281	3.855	3.998	16.13	12.43
1g1cA	0.7790	0.7741	2.717	2.746	18.70	15.05
1gjxA	0.4338	0.3799	7.372	7.953	8.12	9.21
1gnuA	0.5231	0.5528	8.703	9.188	13.88	15.64
1gpt_	0.5204	0.4830	5.591	6.641	10.15	9.32
1gyvA	0.7399	0.7678	3.501	3.435	20.51	17.32
1hbkA	0.6652	0.6586	3.548	3.708	13.98	13.26
1itpA	0.3315	0.3005	11.747	10.892	7.91	9.88
1jnuA	0.7533	0.7466	2.613	2.638	18.10	15.66
1kjs_	0.3866	0.3856	8.550	8.704	9.30	14.10
1kviA	0.7067	0.7067	2.100	2.100	13.03	10.85
1mkyA3	0.4139	0.4026	5.175	5.590	14.18	14.25
1mla_2	0.6153	0.6474	3.140	2.879	12.74	12.89
1mn8A	0.3502	0.3478	7.056	7.481	10.69	10.18
1n0uA4	0.4737	0.4567	4.343	4.638	12.92	11.38
1ne3A	0.4899	0.4281	4.542	6.091	10.79	9.75
1no5A	0.4155	0.3902	10.922	10.918	12.51	15.57
1npsA	0.7732	0.7508	2.254	2.332	14.77	13.76
1o2fB_	0.3640	0.3366	8.661	9.328	7.47	11.08
1of9A	0.5417	0.5400	3.594	3.533	14.73	12.29
1ogwA_	0.6973	0.6973	2.703	2.703	13.90	11.47
1orgA	0.7727	0.7547	2.601	2.682	20.89	15.89
1pgx_	0.5164	0.5637	3.310	3.200	9.38	11.33
1r69_	0.7309	0.7309	1.997	1.997	11.54	10.01
1sfp_	0.7485	0.7360	5.242	5.295	20.68	15.76
1shfA	0.7958	0.8183	1.597	1.494	8.62	11.68
1sro_	0.6341	0.6205	3.613	3.734	13.63	11.72
1ten_	0.7867	0.7867	1.994	1.994	16.72	12.66
1tfi_	0.5151	0.4983	4.613	5.077	10.67	9.87
1thx_	0.8011	0.8043	2.322	2.236	20.17	15.23
1tif_	0.3242	0.3339	7.584	7.574	9.19	9.58
1tig_	0.4245	0.5609	9.576	3.580	9.47	10.76
1vcc_	0.3974	0.3820	6.571	8.170	10.54	10.15
256bA	0.7478	0.7478	3.439	3.439	19.43	16.97
2a0b_	0.7742	0.8123	2.748	2.420	20.64	0.08
2cr7A	0.4067	0.3681	4.890	8.304	8.03	0.08
2f3nA	0.7259	0.7259	1.939	1.939	12.22	10.85
2pcy_	0.6413	0.6454	4.600	4.966	16.35	17.17
2reb_2	0.3316	0.3372	7.011	5.928	6.95	10.88
	Avg. TM-score		Total C_α RMSD		Total CPU time	
	0.574379	0.578386	284.937	286.09	687.45	746.84

Sample size 4000

Target	TM-score		C_α RMSD		CPU Time	
	SPICKER	Calibur	SPICKER	Calibur	SPICKER	Calibur
1abv_	0.2955	0.2793	13.957	12.986	12.77	31.16
1af7__	0.4601	0.4916	4.854	4.379	13.87	22.17
1ah9_	0.4991	0.6450	4.768	3.314	24.56	26.13
1aoy_	0.6693	0.6693	4.766	4.766	30.65	26.71
1b4bA	0.4099	0.4844	6.578	5.684	17.33	28.50
1b72A	0.6641	0.6722	3.141	3.233	11.64	24.34
1bm8_	0.3502	0.3627	7.576	6.842	37.32	29.53
1bq9A	0.3682	0.3855	7.894	7.826	15.25	22.79
1cewI	0.1737	0.1840	16.052	16.137	37.83	38.74
1cqkA	0.8523	0.8523	1.687	1.687	39.39	37.76
1csp_	0.7269	0.7190	2.369	2.384	31.13	30.97
1cy5A	0.8821	0.8821	1.661	1.661	39.05	36.31
1dcjA_	0.3477	0.3701	11.461	12.182	16.68	26.67
1di2A_	0.7747	0.7667	2.408	2.580	27.64	29.97
1dtjA_	0.7821	0.7878	2.180	2.062	33.58	32.19
1egxA	0.7740	0.7740	2.598	2.598	43.46	38.95
1fadA	0.5772	0.5795	3.664	3.636	36.27	33.48
1fo5A	0.5380	0.5380	3.891	3.891	37.61	31.14
1g1cA	0.7794	0.7794	2.648	2.648	43.76	34.41
1gjxA	0.4338	0.4207	7.372	7.513	14.60	23.65
1gnuA	0.5208	0.5428	8.730	9.027	22.78	36.10
1gpt_	0.5156	0.4920	5.711	6.440	19.78	24.39
1gyvA	0.7609	0.7609	3.444	3.444	44.59	39.75
1hbkA	0.6491	0.6491	3.550	3.550	29.24	34.01
1itpA	0.3294	0.3076	11.260	10.809	12.96	21.07
1jnuA	0.7244	0.7481	2.759	2.643	39.79	36.30
1kjs_	0.3757	0.3653	8.433	8.463	17.87	29.83
1kviA	0.7248	0.7001	2.102	2.135	28.73	27.97
1mkyA3	0.4443	0.4026	4.997	5.590	30.90	31.47
1mla_2	0.6153	0.6153	3.140	3.140	26.87	28.64
1mn8A	0.3579	0.3480	7.096	7.452	17.05	34.38
1n0uA4	0.4519	0.4560	4.567	4.622	26.69	39.22
1ne3A	0.4899	0.3904	4.542	5.773	19.83	23.09
1no5A	0.4120	0.3894	10.879	10.656	21.62	34.59
1npsA	0.7547	0.7671	2.424	2.233	29.53	44.60
1o2fB_	0.3640	0.3570	8.661	9.029	12.36	33.32
1of9A	0.5335	0.5416	3.621	3.637	31.54	30.34
1ogwA_	0.6950	0.6950	2.691	2.691	29.13	39.15
1orgA	0.7566	0.7566	2.659	2.659	43.04	54.84
1pgx_	0.5038	0.5060	3.534	3.332	18.83	36.79
1r69_	0.7324	0.7324	1.985	1.985	24.27	27.86
1sfp_	0.7485	0.7337	5.242	5.344	45.19	38.40
1shfA	0.7958	0.8180	1.597	1.519	14.69	38.78
1sro_	0.6483	0.6483	3.542	3.542	31.83	40.48
1ten_	0.7870	0.7870	2.013	2.013	37.99	32.78
1tfi_	0.5149	0.4991	4.735	5.136	24.66	31.82
1thx_	0.7944	0.7944	2.422	2.422	41.33	47.55
1tif_	0.3248	0.3084	7.511	7.871	13.12	29.21
1tig_	0.4685	0.5609	9.690	3.580	16.28	33.84
1vcc_	0.3913	0.3809	6.539	8.190	21.10	30.11
256bA	0.7515	0.7413	3.420	3.462	42.78	51.26
2a0b_	0.7843	0.7893	2.654	2.667	42.25	49.48
2cr7A	0.4793	0.3700	3.642	8.205	11.80	21.51
2f3nA	0.7263	0.7263	1.938	1.938	33.94	26.97
2pcy_	0.6411	0.6424	4.629	4.972	33.14	35.47
2reb_2	0.3322	0.3372	7.069	5.928	11.92	30.40
	Avg. TM-score		Total C_α RMSD		Total CPU time	
	0.576045	0.576859	284.953	284.108	1851.34	1533.81

Sample size 5500

Target	TM-score		C_α RMSD		CPU Time	
	SPICKER	Calibur	SPICKER	Calibur	SPICKER	Calibur
1abv_	0.3010	0.2868	13.955	13.050	16.84	55.96
1af7__	0.4602	0.4767	4.754	4.452	19.41	42.25
1ah9_	0.4670	0.6337	4.810	3.017	41.19	50.79
1aoy_	0.6727	0.6727	4.748	4.748	59.36	51.32
1b4bA	0.4099	0.4840	6.578	5.571	29.87	55.01
1b72A	0.6631	0.6722	3.035	3.233	21.90	47.10
1bm8_	0.4318	0.3580	7.369	7.072	67.66	55.45
1bq9A	0.3786	0.3662	7.890	8.041	27.33	41.38
1cewI	0.1728	0.1840	16.053	16.137	66.06	72.68
1cqkA	0.8622	0.8523	1.658	1.687	74.76	68.67
1csp_	0.7269	0.7190	2.369	2.384	56.61	53.17
1cy5A	0.8701	0.8701	1.620	1.620	70.07	62.93
1dcjA_	0.3467	0.3528	10.575	11.933	26.54	47.94
1di2A_	0.7434	0.7683	2.721	2.620	48.38	53.21
1dtjA_	0.8065	0.8065	1.881	1.881	65.13	56.25
1egxA	0.7876	0.7731	2.457	2.617	82.27	73.54
1fadA	0.5795	0.5773	3.636	3.623	65.75	63.13
1fo5A	0.5250	0.5457	3.987	3.840	68.16	60.22
1g1cA	0.7790	0.7790	2.717	2.717	82.29	66.11
1gjxA	0.4440	0.4104	7.272	7.830	21.79	43.84
1gnuA	0.5243	0.5420	8.711	9.150	40.28	68.02
1gpt_	0.5075	0.5113	5.359	6.292	38.00	45.24
1gyvA	0.7399	0.7692	3.501	3.414	80.01	74.09
1hbkA	0.6725	0.6594	3.474	3.577	52.36	63.42
1itpA	0.3294	0.3076	11.260	10.809	21.57	40.46
1jnuA	0.7244	0.7481	2.759	2.643	69.50	70.89
1kjs_	0.3757	0.3614	8.433	8.558	29.41	58.66
1kviA	0.6965	0.7273	2.246	2.096	64.39	55.34
1mkyA3	0.4139	0.4026	5.175	5.590	52.47	61.02
1mla_2	0.6219	0.6219	3.036	3.036	50.02	56.89
1mn8A	0.3566	0.3566	7.447	7.447	31.59	48.67
1n0uA4	0.4519	0.4571	4.567	4.623	50.56	54.68
1ne3A	0.4189	0.5027	5.579	3.625	44.41	43.15
1no5A	0.4180	0.4251	10.588	10.695	37.15	65.98
1npsA	0.7497	0.7671	2.303	2.233	54.09	61.10
1o2fB_	0.3575	0.3476	8.681	8.941	16.89	46.07
1of9A	0.5335	0.5335	3.621	3.621	62.56	57.98
1ogwA_	0.6950	0.7011	2.691	2.672	59.04	55.77
1orgA	0.7566	0.7566	2.659	2.659	81.97	76.05
1pgx_	0.5043	0.5044	3.506	3.272	34.18	59.71
1r69_	0.7338	0.7338	1.982	1.982	46.30	57.75
1sfp_	0.7462	0.7462	5.312	5.312	86.55	78.67
1shfA	0.7955	0.8158	1.545	1.443	29.68	62.59
1sro_	0.6553	0.6258	3.619	3.756	61.49	63.67
1ten_	0.7881	0.7881	2.011	2.011	69.28	69.34
1tfi_	0.5194	0.4761	4.762	5.102	46.00	55.48
1thx_	0.8043	0.8043	2.236	2.236	77.74	77.72
1tif_	0.3260	0.2862	7.447	9.425	19.85	44.06
1tig_	0.4245	0.5554	9.576	3.577	23.34	49.81
1vcc_	0.3906	0.3796	6.598	8.233	30.55	48.76
256bA	0.7506	0.7506	3.507	3.507	82.21	74.43
2a0b_	0.8029	0.7784	2.502	2.640	77.28	79.55
2cr7A	0.4781	0.3699	3.680	8.239	17.92	47.85
2f3nA	0.7060	0.7060	2.024	2.024	50.61	59.66
2pcy_	0.6411	0.6411	4.629	4.629	60.67	73.77
2reb_2	0.3322	0.3445	7.069	5.913	20.26	49.11
	Avg. TM-score		Total C_α RMSD		Total CPU time	
	0.574475	0.57845	284.18	283.055	3276.36	2781.55

Sample size 7000

Target	TM-score		C_α RMSD		CPU Time	
	SPICKER	Calibur	SPICKER	Calibur	SPICKER	Calibur
1abv_	0.2972	0.2883	13.966	12.999	27.21	90.39
1af7__	0.4601	0.4791	4.854	4.428	36.63	67.91
1ah9_	0.5006	0.6450	4.756	3.314	65.81	87.39
1aoy_	0.6601	0.6484	4.584	4.977	97.61	88.74
1b4bA	0.4099	0.4728	6.578	5.751	42.94	93.53
1b72A	0.6659	0.6764	3.164	3.102	30.19	83.40
1bm8_	0.3502	0.3627	7.576	6.842	105.48	91.92
1bq9A	0.3781	0.3662	7.838	8.041	39.78	70.40
1cewI	0.1719	0.1840	16.049	16.137	107.29	113.99
1cqkA	0.8540	0.8328	1.716	1.844	120.82	114.11
1csp_	0.7252	0.7187	2.355	2.314	93.34	92.55
1cy5A	0.8697	0.8697	1.613	1.613	108.84	105.90
1dcjA_	0.3454	0.3751	11.651	12.145	38.87	84.05
1di2A_	0.7690	0.7668	2.504	2.618	80.48	94.59
1dtjA_	0.7622	0.7800	2.380	2.234	109.18	91.15
1egxA	0.7779	0.7745	2.526	2.606	133.55	122.15
1fadA	0.5795	0.5773	3.636	3.623	111.26	106.61
1fo5A	0.5250	0.5442	3.987	3.879	113.44	100.26
1g1cA	0.7812	0.7812	2.499	2.499	129.05	112.68
1gjxA	0.4368	0.4104	7.395	7.830	35.67	76.07
1gnuA	0.5218	0.5420	8.707	9.150	60.37	115.32
1gpt_	0.5204	0.4736	5.591	6.296	62.51	76.34
1gyvA	0.7609	0.7609	3.444	3.444	123.56	122.74
1hbkA	0.6501	0.6501	3.633	3.633	75.48	105.53
1itpA	0.3419	0.3076	11.258	10.809	30.06	67.60
1jnuA	0.7601	0.7506	2.575	2.681	110.12	117.63
1kjs_	0.3828	0.3653	8.489	8.463	44.04	96.92
1kviA	0.6965	0.7067	2.246	2.100	77.38	92.48
1mkyA3	0.4139	0.4095	5.175	5.579	62.97	74.00
1mla_2	0.6153	0.6182	3.140	3.079	86.82	93.05
1mn8A	0.3584	0.3532	7.053	7.009	48.95	76.22
1n0uA4	0.4737	0.4560	4.343	4.622	80.85	89.87
1ne3A	0.4899	0.5214	4.542	4.073	72.46	72.60
1no5A	0.4125	0.4251	10.920	10.695	59.00	108.71
1npsA	0.7565	0.7565	2.280	2.280	84.75	104.89
1o2fB_	0.3592	0.3943	8.688	5.524	33.17	75.06
1of9A	0.5329	0.5355	3.644	3.631	97.56	95.93
1ogwA_	0.6827	0.6827	2.739	2.739	90.08	92.98
1orgA	0.7566	0.7566	2.659	2.659	123.62	122.48
1pgx_	0.5043	0.5043	3.506	3.506	48.31	267.43
1r69_	0.7338	0.7338	1.982	1.982	63.07	263.71
1sfp_	0.7353	0.7249	5.224	5.298	128.68	441.78
1shfA	0.8045	0.8255	1.561	1.476	40.81	266.36
1sro_	0.6483	0.6483	3.542	3.542	89.93	299.72
1ten_	0.7927	0.7927	1.930	1.930	106.30	355.81
1tfi_	0.5526	0.4889	4.975	4.746	80.78	221.62
1thx_	0.7867	0.8014	2.407	2.288	125.32	431.31
1tif_	0.3248	0.3180	7.511	7.783	32.90	72.57
1tig_	0.4685	0.5609	9.690	3.580	37.33	79.62
1vcc_	0.3906	0.3760	6.598	8.128	48.15	73.71
256bA	0.7600	0.7585	3.448	3.438	127.43	113.28
2a0b_	0.7843	0.7745	2.654	2.782	119.38	119.19
2cr7A	0.4983	0.3668	3.673	8.220	25.40	67.46
2f3nA	0.7188	0.7261	1.960	1.936	74.61	83.87
2pcy_	0.6411	0.6454	4.629	4.966	91.98	112.93
2reb_2	0.3235	0.3487	6.884	5.906	29.38	70.58
	Avg. TM-score		Total C_α RMSD		Total CPU time	
	0.576323	0.578823	284.927	278.769	7029.09	4320.95

Sample size 8500

Target	TM-score		C_α RMSD		CPU Time	
	SPICKER	Calibur	SPICKER	Calibur	SPICKER	Calibur
1abv_	0.2975	0.2730	13.987	13.126	34.12	139.88
1af7__	0.4601	0.4767	4.854	4.452	40.14	109.58
1ah9_	0.4991	0.6327	4.768	3.002	69.49	132.28
1aoy_	0.6603	0.6603	4.828	4.828	135.15	133.27
1b4bA	0.4099	0.4728	6.578	5.751	58.02	143.38
1b72A	0.6659	0.6764	3.164	3.102	40.66	126.38
1bm8_	0.4318	0.3627	7.369	6.842	148.62	142.11
1bq9A	0.3939	0.3734	7.800	8.181	52.37	112.83
1cewI	0.1764	0.1867	16.072	16.129	140.38	176.60
1cqkA	0.8512	0.8328	1.756	1.844	161.81	168.06
1csp_	0.7269	0.7190	2.369	2.384	127.71	129.38
1cy5A	0.8779	0.8779	1.660	1.660	155.86	154.23
1dcjA_	0.3789	0.3735	10.510	12.204	53.56	117.72
1di2A_	0.7989	0.7684	2.336	2.627	103.39	128.48
1dtjA_	0.8065	0.8065	1.881	1.881	144.60	161.20
1egxA	0.7740	0.7740	2.598	2.598	181.12	194.11
1fadA	0.5772	0.5772	3.664	3.664	152.74	173.96
1fo5A	0.5301	0.5440	3.947	3.845	153.42	167.70
1g1cA	0.7753	0.7769	2.736	2.719	183.50	175.74
1gjxA	0.4558	0.3852	7.318	7.644	43.93	125.74
1gnuA	0.5218	0.5539	8.707	9.183	78.96	174.33
1gpt_	0.5075	0.5113	5.359	6.292	80.37	139.12
1gyvA	0.7399	0.7692	3.501	3.414	172.17	186.83
1hbkA	0.6633	0.6633	3.482	3.482	104.44	169.41
1itpA	0.3294	0.3076	11.260	10.809	43.20	102.15
1jnuA	0.7266	0.7489	2.854	2.576	150.75	175.65
1kjs_	0.3830	0.3608	8.436	8.511	61.23	158.56
1kviA	0.6994	0.7067	2.255	2.100	112.88	139.61
1mkyA3	0.4139	0.4095	5.175	5.579	61.71	74.74
1mla_2	0.6219	0.6219	3.036	3.036	110.36	149.94
1mn8A	0.3528	0.3532	7.077	7.009	66.00	110.70
1n0uA4	0.4519	0.4571	4.567	4.623	111.36	130.11
1ne3A	0.4296	0.4315	5.533	5.915	78.68	122.68
1no5A	0.4180	0.4251	10.588	10.695	80.33	172.91
1npsA	0.7565	0.7565	2.280	2.280	115.91	166.41
1o2fB_	0.3592	0.3929	8.688	5.541	43.82	123.12
1of9A	0.5393	0.5393	3.603	3.603	136.86	156.99
1ogwA_	0.6950	0.7011	2.691	2.672	127.69	156.48
1orgA	0.7537	0.7537	2.694	2.694	172.08	196.87
1pgx_	0.5038	0.5044	3.523	3.272	63.55	151.03
1r69_	0.7538	0.7538	1.971	1.971	92.90	126.61
1sfp_	0.7455	0.7455	5.261	5.261	193.17	171.35
1shfA	0.8256	0.8183	1.369	1.494	62.54	126.16
1sro_	0.6483	0.6483	3.542	3.542	133.66	129.75
1ten_	0.8233	0.8233	1.837	1.837	153.79	152.14
1tfi_	0.5151	0.4983	4.613	5.077	107.59	135.07
1thx_	0.8043	0.8014	2.236	2.288	172.92	163.90
1tif_	0.3260	0.3339	7.447	7.574	40.63	103.66
1tig_	0.4245	0.5609	9.576	3.580	49.04	116.63
1vcc_	0.4026	0.3765	6.507	8.164	71.34	119.60
256bA	0.7600	0.7600	3.448	3.448	180.68	178.10
2a0b_	0.7843	0.8051	2.654	2.470	167.48	190.68
2cr7A	0.4793	0.3717	3.642	8.270	37.83	115.55
2f3nA	0.7259	0.7259	1.939	1.939	111.31	140.30
2pcy_	0.6273	0.6402	4.710	4.658	137.62	173.34
2reb_2	0.3322	0.3487	7.069	5.906	41.17	119.79
	Avg. TM-score		Total C_α RMSD		Total CPU time	
	0.57843	0.580889	283.325	279.248	8132.9	5906.61

Sample size 10000

Target	TM-score		C_α RMSD		CPU Time	
	SPICKER	Calibur	SPICKER	Calibur	SPICKER	Calibur
1abv_	0.2955	0.2868	13.957	13.050	41.15	188.74
1af7__	0.4601	0.4767	4.854	4.452	48.16	142.39
1ah9_	0.5112	0.6644	4.658	3.083	119.12	177.62
1aoy_	0.6726	0.6726	4.801	4.801	184.83	180.65
1b4bA	0.4068	0.4728	6.439	5.751	75.03	186.21
1b72A	0.6659	0.6722	3.164	3.233	52.82	155.72
1bm8_	0.4318	0.3580	7.369	7.072	192.26	190.36
1bq9A	0.3688	0.3789	7.858	8.221	67.29	170.25
1cewI	0.1728	0.1798	16.053	16.207	186.78	244.90
1cqkA	0.8589	0.8523	1.668	1.687	219.41	228.70
1csp_	0.7252	0.7151	2.355	2.292	170.84	197.70
1cy5A	0.8779	0.8779	1.660	1.660	208.94	226.97
1dcjA_	0.3526	0.3701	10.786	12.182	69.04	160.54
1di2A_	0.7747	0.7668	2.408	2.618	133.49	182.21
1dtjA_	0.7821	0.7960	2.180	2.115	195.78	183.68
1egxA	0.7740	0.7740	2.598	2.598	242.45	253.36
1fadA	0.5772	0.5800	3.664	3.620	209.42	229.03
1fo5A	0.5360	0.5457	3.907	3.840	201.01	212.22
1g1cA	0.7794	0.7794	2.648	2.648	245.22	229.49
1gjxA	0.4558	0.4104	7.318	7.830	54.79	159.72
1gnuA	0.5218	0.5446	8.707	9.000	97.25	219.25
1gpt_	0.5287	0.4979	5.311	6.324	104.87	158.53
1gyvA	0.7609	0.7692	3.444	3.414	227.96	245.03
1hbkA	0.6662	0.6501	3.473	3.633	134.93	225.31
1itpA	0.3294	0.3207	11.260	10.924	53.41	144.63
1jnuA	0.7601	0.7506	2.575	2.681	200.64	234.00
1kjs_	0.3830	0.3608	8.436	8.511	78.28	204.28
1kviA	0.6930	0.7001	2.322	2.135	147.71	188.20
1mkyA3	0.4139	0.4095	5.175	5.579	58.68	73.61
1mla_2	0.6153	0.6349	3.140	2.824	147.39	177.27
1mn8A	0.3528	0.3480	7.077	7.452	79.34	165.45
1n0uA4	0.4737	0.4571	4.343	4.623	148.65	188.43
1ne3A	0.4899	0.5214	4.542	4.073	129.51	152.19
1no5A	0.4125	0.4251	10.920	10.695	103.73	221.15
1npsA	0.7565	0.7565	2.280	2.280	152.01	210.45
1o2fB_	0.3592	0.3955	8.688	5.569	55.03	141.68
1of9A	0.5335	0.5335	3.621	3.621	183.40	208.57
1ogwA_	0.6844	0.7011	2.717	2.672	169.14	180.37
1orgA	0.7566	0.7566	2.659	2.659	238.77	259.97
1pgx_	0.5043	0.5043	3.506	3.506	89.11	193.91
1r69_	0.7338	0.7338	1.982	1.982	125.73	191.24
1sfp_	0.7485	0.7281	5.242	5.326	253.94	238.10
1shfA	0.7887	0.8183	1.550	1.494	76.17	169.65
1sro_	0.6341	0.6341	3.613	3.613	177.05	195.75
1ten_	0.7927	0.7927	1.930	1.930	203.16	219.03
1tfi_	0.5146	0.4761	4.902	5.102	142.18	173.68
1thx_	0.7844	0.7844	2.462	2.462	228.14	245.90
1tif_	0.3260	0.3274	7.447	7.739	52.82	165.20
1tig_	0.4245	0.5636	9.576	3.541	60.75	173.20
1vcc_	0.3924	0.3717	6.616	8.159	89.31	141.52
256bA	0.7600	0.7600	3.448	3.448	237.88	225.38
2a0b_	0.8029	0.8065	2.502	2.428	225.06	246.68
2cr7A	0.4793	0.3699	3.642	8.239	46.61	137.52
2f3nA	0.7263	0.7263	1.938	1.938	151.26	174.07
2pcy_	0.6357	0.6402	4.644	4.658	183.35	219.61
2reb_2	0.3235	0.3487	6.884	5.906	51.70	136.18
	Avg. TM-score		Total C_α RMSD		Total CPU time	
	0.577543	0.581236	282.919	279.1	10745.5	7822.75

Sample size 11500

Target	TM-score		C_α RMSD		CPU Time	
	SPICKER	Calibur	SPICKER	Calibur	SPICKER	Calibur
1abv_	0.2955	0.2868	13.957	13.050	53.50	248.72
1af7__	0.4601	0.4767	4.854	4.452	60.01	181.75
1ah9_	0.5082	0.6337	4.701	3.017	146.34	215.75
1aoy_	0.6692	0.6692	4.752	4.752	237.59	229.88
1b4bA	0.4099	0.4728	6.578	5.751	100.57	237.42
1b72A	0.6641	0.6722	3.141	3.233	69.91	197.42
1bm8_	0.4318	0.3627	7.369	6.842	260.92	250.95
1bq9A	0.3688	0.3734	7.858	8.181	86.36	217.78
1cewI	0.1728	0.1840	16.053	16.137	244.65	347.99
1cqkA	0.8512	0.8523	1.756	1.687	288.82	335.10
1csp_	0.7252	0.7190	2.355	2.384	221.88	262.33
1cy5A	0.8779	0.8779	1.660	1.660	271.22	315.64
1dcjA_	0.3542	0.3751	10.798	12.145	95.10	235.30
1di2A_	0.7989	0.7668	2.336	2.618	174.36	227.02
1dtjA_	0.8065	0.7960	1.881	2.115	264.63	247.94
1egxA	0.7740	0.7740	2.598	2.598	319.18	350.75
1fadA	0.5772	0.5800	3.664	3.620	274.16	269.38
1fo5A	0.5313	0.5457	3.940	3.840	276.59	260.63
1g1cA	0.7794	0.7794	2.648	2.648	328.53	288.74
1gjxA	0.4440	0.4104	7.272	7.830	74.22	214.38
1gnuA	0.5218	0.5420	8.707	9.150	132.67	308.52
1gpt_	0.5182	0.5113	5.525	6.292	139.28	236.08
1gyvA	0.7609	0.7641	3.444	3.408	299.84	346.65
1hbkA	0.6633	0.6633	3.482	3.482	180.49	291.51
1itpA	0.3419	0.3076	11.258	10.809	69.03	168.11
1jnuA	0.7522	0.7479	2.599	2.635	269.65	329.17
1kjs_	0.3829	0.3608	8.430	8.511	102.94	250.30
1kviA	0.6994	0.7155	2.255	2.051	198.28	258.87
1mkyA3	0.4139	0.4095	5.175	5.579	61.45	74.24
1mla_2	0.6219	0.6349	3.036	2.824	193.81	235.98
1mn8A	0.3584	0.3480	7.053	7.452	111.01	200.40
1n0uA4	0.4519	0.4571	4.567	4.623	206.14	225.88
1ne3A	0.4899	0.5214	4.542	4.073	188.26	206.39
1no5A	0.4125	0.4251	10.920	10.695	134.71	305.98
1npsA	0.7702	0.7700	2.166	2.223	205.14	305.67
1o2fB_	0.3592	0.3585	8.688	9.004	57.49	194.54
1of9A	0.5335	0.5380	3.621	3.583	247.22	265.44
1ogwA_	0.6827	0.6827	2.739	2.739	224.96	268.64
1orgA	0.7566	0.7566	2.659	2.659	314.46	337.62
1pgx_	0.5038	0.5060	3.523	3.332	119.87	248.88
1r69_	0.7538	0.7538	1.971	1.971	162.14	247.92
1sfp_	0.7455	0.7455	5.261	5.261	346.90	338.69
1shfA	0.7887	0.8146	1.550	1.487	103.47	243.17
1sro_	0.6483	0.6483	3.542	3.542	239.55	227.31
1ten_	0.8233	0.8233	1.837	1.837	275.25	284.47
1tfi_	0.5149	0.4761	4.735	5.102	194.70	205.91
1thx_	0.7867	0.7966	2.407	2.262	305.69	298.01
1tif_	0.3260	0.3339	7.447	7.574	69.12	198.28
1tig_	0.4245	0.5609	9.576	3.580	82.37	218.23
1vcc_	0.4026	0.3760	6.507	8.128	117.84	207.58
256bA	0.7506	0.7506	3.507	3.507	323.91	321.58
2a0b_	0.7933	0.8069	2.570	2.485	287.50	341.70
2cr7A	0.4793	0.3717	3.642	8.270	57.36	199.80
2f3nA	0.7263	0.7263	1.938	1.938	194.30	248.38
2pcy_	0.6273	0.6402	4.710	4.658	240.03	313.47
2reb_2	0.3235	0.3487	6.884	5.906	68.83	205.59
	Avg. TM-score		Total C_α RMSD		Total CPU time	
	0.578748	0.582175	282.644	281.192	14293.8	10374.2

Sample size 13000

Target	TM-score		C_α RMSD		CPU Time	
	SPICKER	Calibur	SPICKER	Calibur	SPICKER	Calibur
1abv_	0.2903	0.2868	13.941	13.050	56.59	275.00
1af7__	0.4654	0.4767	4.728	4.452	68.00	209.90
1ah9_	0.5082	0.6313	4.701	3.341	196.37	309.03
1aoy_	0.6598	0.6598	4.761	4.761	293.26	305.84
1b4bA	0.4099	0.4840	6.578	5.571	115.83	275.50
1b72A	0.6651	0.6722	3.195	3.233	83.21	234.53
1bm8_	0.4317	0.3580	7.384	7.072	330.91	328.53
1bq9A	0.3781	0.3696	7.838	8.140	122.94	256.03
1cewI	0.1728	0.1840	16.053	16.137	302.90	144.22
1cqkA	0.8210	0.8523	1.946	1.687	347.84	373.72
1csp_	0.7269	0.7190	2.369	2.384	248.54	281.37
1cy5A	0.8739	0.8779	1.573	1.660	332.17	362.15
1dcjA_	0.3542	0.3751	10.798	12.145	139.39	289.45
1di2A_	0.7989	0.7683	2.336	2.620	210.09	293.51
1dtjA_	0.7821	0.7960	2.180	2.115	336.15	305.77
1egxA	0.7740	0.7731	2.598	2.617	392.68	410.27
1fadA	0.5784	0.5800	3.611	3.620	307.85	342.60
1fo5A	0.5197	0.5442	3.970	3.879	335.91	358.39
1g1cA	0.7794	0.7812	2.648	2.499	401.02	377.20
1gjxA	0.4338	0.4104	7.372	7.830	83.97	221.46
1gnuA	0.5202	0.5420	8.820	9.150	174.49	363.54
1gpt_	0.5207	0.4979	5.467	6.324	167.84	265.71
1gyvA	0.7609	0.7641	3.444	3.408	300.66	321.79
1hbkA	0.6633	0.6633	3.482	3.482	221.08	340.59
1itpA	0.3268	0.3076	11.699	10.809	77.15	231.61
1jnuA	0.7242	0.7506	2.830	2.681	318.90	386.69
1kjs_	0.3789	0.3614	8.466	8.558	130.61	338.14
1kviA	0.7059	0.7067	2.153	2.100	235.37	312.38
1mkyA3	0.4307	0.4095	5.095	5.579	58.96	80.75
1mla_2	0.6219	0.6219	3.036	3.036	217.99	271.54
1mn8A	0.3579	0.3480	7.096	7.452	133.32	248.44
1n0uA4	0.4645	0.4571	4.460	4.623	217.24	282.25
1ne3A	0.4899	0.5214	4.542	4.073	198.11	138.25
1no5A	0.4456	0.4251	10.832	10.695	152.46	332.10
1npsA	0.7686	0.7671	2.287	2.233	237.88	350.80
1o2fB_	0.3738	0.3585	8.345	9.004	64.95	240.68
1of9A	0.5391	0.5422	3.616	3.635	292.49	323.60
1ogwA_	0.6606	0.7011	2.867	2.672	269.10	302.23
1orgA	0.7666	0.7566	2.583	2.659	383.66	418.19
1pgx_	0.5043	0.5295	3.506	3.260	140.25	139.22
1r69_	0.7381	0.7538	2.027	1.971	197.81	282.05
1sfp_	0.7462	0.7281	5.312	5.326	424.36	409.11
1shfA	0.8123	0.8158	1.471	1.443	143.46	295.98
1sro_	0.6616	0.6483	3.571	3.542	289.61	305.25
1ten_	0.7927	0.8233	1.930	1.837	327.79	335.17
1tfi_	0.4889	0.4991	4.746	5.136	238.42	254.32
1thx_	0.7944	0.7966	2.422	2.262	367.48	404.26
1tif_	0.3222	0.3339	7.584	7.574	74.72	256.24
1tig_	0.4685	0.5609	9.690	3.580	93.82	243.61
1vcc_	0.4026	0.3760	6.507	8.128	134.83	249.69
256bA	0.7600	0.7600	3.448	3.448	397.42	397.25
2a0b_	0.7920	0.8034	2.617	2.467	362.17	416.90
2cr7A	0.4879	0.3699	3.605	8.239	65.70	210.67
2f3nA	0.7210	0.7263	1.946	1.938	235.38	284.82
2pcy_	0.6273	0.6402	4.710	4.658	293.46	367.57
2reb_2	0.3285	0.3487	7.003	5.906	75.90	252.92
	Avg. TM-score		Total C_α RMSD		Total CPU time	
	0.578432	0.582425	283.795	281.701	16608.8	12420.5
