# Supplementary material for: Calibur: a tool for clustering large numbers of protein decoys
Source: BMC Bioinformatics. 2010 Jan 13;11:25. doi: 10.1186/1471-2105-11-25 (PMC2881085; doi:10.1186/1471-2105-11-25)
Supplement: Additional file 2 — Details of experiments using sample sets of sizes 16000, 19000, 22000, 25000, 28000 and 31000. [file 1471-2105-11-25-S2.pdf]

Calibur's CPU time and obtained decoy scores using 16000 decoys

Target	TM-score	C_α RMSD	CPU Time
1abv_	0.2868	13.050	57.88
1af7__	0.4916	4.379	68.39
1ah9_	0.6313	3.341	420.82
1aoy_	0.6598	4.761	830.93
1b4bA	0.4840	5.571	119.30
1b72A	0.6722	3.233	81.74
1bm8_	0.3580	7.072	827.47
1bq9A	0.3734	8.181	253.30
1cewI	0.1840	16.137	781.12
1cqkA	0.8523	1.687	993.67
1csp_	0.7190	2.384	252.52
1cy5A	0.8779	1.660	937.30
1dcjA_	0.3701	12.182	265.42
1di2A_	0.7683	2.620	449.80
1dtjA_	0.8065	1.881	893.01
1egxA	0.7740	2.598	1198.61
1fadA	0.5800	3.620	311.07
1fo5A	0.5432	3.871	974.06
1g1cA	0.7794	2.648	1312.40
1gjxA	0.4104	7.830	86.92
1gnuA	0.5420	9.150	377.14
1gpt_	0.5207	6.445	374.16
1gyvA	0.7678	3.435	297.59
1hbkA	0.6633	3.482	566.85
1itpA	0.3076	10.809	78.42
1jnuA	0.7506	2.681	859.70
1kjs_	0.3608	8.511	298.73
1kviA	0.7067	2.100	619.23
1mkyA3	0.4095	5.579	62.56
1mla_2	0.6349	2.824	224.51
1mn8A	0.3480	7.452	129.24
1n0uA4	0.4571	4.623	216.24
1ne3A	0.4281	6.091	145.67
1no5A	0.4251	10.695	157.41
1npsA	0.7565	2.280	595.34
1o2fB_	0.3585	9.004	69.83
1of9A	0.5422	3.635	817.08
1ogwA_	0.6844	2.717	745.96
1orgA	0.7666	2.583	1056.22
1pgx_	0.5295	3.260	354.70
1r69_	0.7538	1.971	483.21
1sfp_	0.7455	5.261	1300.52
1shfA	0.8183	1.494	250.25
1sro_	0.6483	3.542	768.01
1ten_	0.8202	1.799	977.52
1tfi_	0.4991	5.136	574.84
1thx_	0.7966	2.262	1044.96
1tif_	0.3180	7.783	76.79
1tig_	0.5609	3.580	96.74
1vcc_	0.3717	8.159	351.04
256bA	0.7600	3.448	1229.45
2a0b_	0.8034	2.467	992.32
2cr7A	0.3699	8.239	68.50
2f3nA	0.7188	1.960	613.22
2pcy_	0.6402	4.658	791.07
2reb_2	0.3372	5.928	76.41
	Avg. TM-score	Total C_α RMSD	Total CPU time
	0.581143	283.749	28857.2

Calibur's CPU time and obtained decoy scores using 19000 decoys

Target	TM-score	C_α RMSD	CPU Time
1abv_	0.2868	13.050	60.96
1af7__	0.4767	4.452	71.04
1ah9_	0.6450	3.314	531.55
1aoy_	0.6598	4.761	1166.59
1b4bA	0.4728	5.751	123.13
1b72A	0.6722	3.233	84.60
1bm8_	0.3580	7.072	1186.99
1bq9A	0.3734	8.181	340.64
1cewI	0.1840	16.137	1100.84
1cqkA	0.8523	1.687	1362.21
1csp_	0.7190	2.384	249.38
1cy5A	0.8779	1.660	1296.45
1dcjA_	0.3701	12.182	375.08
1di2A_	0.7668	2.618	614.03
1dtjA_	0.7960	2.115	1206.60
1egxA	0.7740	2.598	1564.89
1fadA	0.5800	3.620	310.15
1fo5A	0.5432	3.871	1330.68
1g1cA	0.7794	2.648	1834.58
1gjxA	0.4104	7.830	87.10
1gnuA	0.5420	9.150	539.13
1gpt_	0.5113	6.292	507.13
1gyvA	0.7641	3.408	297.17
1hbkA	0.6633	3.482	786.94
1itpA	0.3076	10.809	80.02
1jnuA	0.7506	2.681	1201.97
1kjs_	0.3830	8.436	435.27
1kviA	0.7067	2.100	873.41
1mla_2	0.6349	2.824	63.96
1mkyA3	0.4037	5.531	226.02
1mn8A	0.3566	7.447	137.44
1n0uA4	0.4571	4.623	222.04
1ne3A	0.5214	4.073	192.91
1no5A	0.4251	10.695	153.13
1npsA	0.7565	2.280	809.54
1o2fB_	0.3585	9.004	69.68
1of9A	0.5422	3.635	1122.39
1ogwA_	0.6844	2.717	1042.38
1orgA	0.7566	2.659	1497.53
1pgx_	0.5043	3.506	457.16
1r69_	0.7538	1.971	682.84
1sfp_	0.7455	5.261	1823.54
1shfA	0.8183	1.494	350.81
1sro_	0.6483	3.542	1066.59
1ten_	0.8233	1.837	1368.14
1tfi_	0.4889	4.746	809.26
1thx_	0.7966	2.262	1463.04
1tif_	0.3339	7.574	78.61
1tig_	0.5609	3.580	98.25
1vcc_	0.3760	8.128	452.45
2a0b_	0.8034	2.467	1646.85
2cr7A	0.3699	8.239	1381.10
2f3nA	0.7263	1.938	71.61
2pcy_	0.6402	4.658	842.35
2reb_2	0.3372	5.928	1101.90
256bA	0.7600	3.448	78.86
	Avg. TM-score	Total C_α RMSD	Total CPU time
	0.582325	281.589	38928.9

Calibur's CPU time and obtained decoy scores using 22000 decoys

Target	TM-score	C_α RMSD	CPU Time
1abv_	0.2868	13.050	69.64
1af7__	0.4767	4.452	76.30
1ah9_	0.6337	3.017	820.47
1aoy_	0.6649	4.724	1622.57
1b4bA	0.4840	5.571	120.92
1b72A	0.6764	3.102	101.11
1bm8_	0.3580	7.072	1435.58
1bq9A	0.3734	8.181	478.05
1cewI	0.1840	16.137	1263.05
1cqkA	0.8523	1.687	1541.70
1csp_	0.7190	2.384	269.94
1cy5A	0.8701	1.620	1718.17
1dcjA_	0.3701	12.182	462.90
1di2A_	0.7668	2.618	685.75
1dtjA_	0.7960	2.115	1352.50
1egxA	0.7740	2.598	1713.56
1fadA	0.5800	3.620	317.96
1fo5A	0.5432	3.871	1597.84
1g1cA	0.7794	2.648	2014.83
1gjxA	0.3926	8.226	100.87
1gnuA	0.5420	9.150	589.29
1gpt_	0.5113	6.292	775.22
1gyvA	0.7678	3.435	296.45
1hbkA	0.6633	3.482	880.96
1itpA	0.3076	10.809	81.66
1jnuA	0.7506	2.681	1311.19
1kjs_	0.3608	8.511	488.67
1kviA	0.7067	2.100	959.85
1mla_2	0.6349	2.824	66.79
1mkyA3	0.4037	5.531	221.07
1mn8A	0.3532	7.009	142.87
1n0uA4	0.4571	4.623	228.53
1ne3A	0.4315	5.915	181.57
1no5A	0.4251	10.695	160.85
1npsA	0.7565	2.280	939.44
1o2fB_	0.3955	5.569	77.81
1of9A	0.5422	3.635	1254.91
1ogwA_	0.6827	2.739	1182.19
1orgA	0.7566	2.659	1653.70
1pgx_	0.5295	3.260	537.23
1r69_	0.7538	1.971	772.03
1sfp_	0.7455	5.261	2074.76
1shfA	0.8183	1.494	460.69
1sro_	0.6483	3.542	1188.50
1ten_	0.8233	1.837	1498.52
1tfi_	0.4991	5.136	1186.52
1thx_	0.8014	2.288	1987.43
1tif_	0.3339	7.574	74.74
1tig_	0.5609	3.580	95.09
1vcc_	0.3717	8.159	560.82
2a0b_	0.7745	2.782	2030.56
2cr7A	0.3699	8.239	1840.51
2f3nA	0.7263	1.938	71.25
2pcy_	0.6402	4.658	936.07
2reb_2	0.3487	5.906	1263.50
256bA	0.7600	3.448	81.01
	Avg. TM-score	Total C_α RMSD	Total CPU time
	0.580996	279.887	45916

Calibur's CPU time and obtained decoy scores using 25000 decoys

Target	TM-score	C_α RMSD	CPU Time
1abv_	0.2897	13.075	63.60
1af7__	0.4767	4.452	74.01
1ah9_	0.6450	3.314	982.94
1aoy_	0.6598	4.761	1949.62
1b4bA	0.4840	5.571	123.14
1b72A	0.6722	3.233	85.51
1bm8_	0.3580	7.072	1384.84
1bq9A	0.3734	8.181	440.22
1cewI	0.1840	16.137	1208.81
1cqkA	0.8523	1.687	1534.90
1csp_	0.7190	2.384	251.45
1cy5A	0.8701	1.620	2209.12
1dcjA_	0.3701	12.182	477.26
1di2A_	0.7668	2.618	685.01
1dtjA_	0.7960	2.115	1341.46
1egxA	0.7740	2.598	1722.85
1fadA	0.5772	3.664	312.90
1fo5A	0.5432	3.871	1468.92
1g1cA	0.7794	2.648	2015.77
1gjxA	0.4104	7.830	88.18
1gnuA	0.5420	9.150	521.99
1gpt_	0.5113	6.292	848.16
1gyvA	0.7678	3.435	294.76
1hbkA	0.6633	3.482	852.60
1itpA	0.3076	10.809	80.19
1jnuA	0.7506	2.681	1312.75
1kjs_	0.3830	8.436	490.65
1kviA	0.7067	2.100	955.61
1mla_2	0.6349	2.824	63.46
1mkyA3	0.4095	5.579	227.24
1mn8A	0.3532	7.009	133.95
1n0uA4	0.4571	4.623	216.03
1ne3A	0.4281	6.091	147.88
1no5A	0.4251	10.695	158.41
1npsA	0.7565	2.280	887.64
1o2fB_	0.3585	9.004	69.11
1of9A	0.5422	3.635	1236.51
1ogwA_	0.6827	2.739	1163.07
1orgA	0.7566	2.659	1636.94
1pgx_	0.5295	3.260	522.97
1r69_	0.7538	1.971	747.93
1sfp_	0.7455	5.261	1999.28
1shfA	0.8183	1.494	417.48
1sro_	0.6483	3.542	1192.94
1ten_	0.8233	1.837	1497.61
1tfi_	0.4983	5.077	1312.53
1thx_	0.8014	2.288	2455.55
1tif_	0.3339	7.574	80.03
1tig_	0.5609	3.580	98.42
1vcc_	0.3760	8.128	528.77
2a0b_	0.8034	2.467	1811.03
2cr7A	0.3699	8.239	2334.31
2f3nA	0.7263	1.938	71.23
2pcy_	0.6425	4.981	952.97
2reb_2	0.3372	5.928	1261.33
256bA	0.7600	3.448	81.27
	Avg. TM-score	Total C_α RMSD	Total CPU time
	0.581545	283.549	47083.1

Calibur's CPU time and obtained decoy scores using 28000 decoys

Target	TM-score	C_α RMSD	CPU Time
1abv_	0.2868	13.050	63.49
1af7__	0.4767	4.452	75.60
1ah9_	0.6450	3.314	1109.40
1aoy_	0.6598	4.761	2419.53
1b4bA	0.4840	5.571	118.25
1b72A	0.6722	3.233	84.22
1bm8_	0.3580	7.072	1392.79
1bq9A	0.3734	8.181	451.99
1cewI	0.1840	16.137	1219.16
1cqkA	0.8523	1.687	1544.21
1csp_	0.7190	2.384	276.55
1cy5A	0.8701	1.620	2781.78
1dcjA_	0.3701	12.182	477.20
1di2A_	0.7668	2.618	673.79
1dtjA_	0.7960	2.115	1331.68
1egxA	0.7740	2.598	1752.23
1fadA	0.5800	3.620	321.82
1fo5A	0.5432	3.871	1486.41
1g1cA	0.7794	2.648	2015.71
1gjxA	0.4104	7.830	83.11
1gnuA	0.5420	9.150	570.49
1gpt_	0.5113	6.292	1072.82
1gyvA	0.7641	3.408	305.42
1hbkA	0.6633	3.482	871.85
1itpA	0.3076	10.809	77.54
1jnuA	0.7506	2.681	1321.04
1kjs_	0.3830	8.436	499.68
1kviA	0.7067	2.100	960.55
1mla_2	0.6349	2.824	62.77
1mkyA3	0.4139	5.175	218.55
1mn8A	0.3480	7.452	133.19
1n0uA4	0.4571	4.623	214.87
1ne3A	0.5214	4.073	205.65
1no5A	0.4251	10.695	154.94
1npsA	0.7565	2.280	968.67
1o2fB_	0.3943	5.524	80.29
1of9A	0.5422	3.635	1238.20
1ogwA_	0.6844	2.717	1138.62
1orgA	0.7566	2.659	1649.84
1pgx_	0.5043	3.506	548.35
1r69_	0.7538	1.971	749.55
1sfp_	0.7455	5.261	2006.09
1shfA	0.8183	1.494	419.59
1sro_	0.6483	3.542	1186.91
1ten_	0.8233	1.837	1531.45
1tfi_	0.4991	5.136	1757.60
1thx_	0.7966	2.262	3200.67
1tif_	0.3339	7.574	79.26
1tig_	0.5609	3.580	97.13
1vcc_	0.3760	8.128	530.03
2a0b_	0.7745	2.782	1840.83
2cr7A	0.3699	8.239	2982.38
2f3nA	0.7263	1.938	66.47
2pcy_	0.6402	4.658	930.52
2reb_2	0.3372	5.928	1241.74
256bA	0.7600	3.448	76.95
	Avg. TM-score	Total C_α RMSD	Total CPU time
	0.58272	278.243	50669.4

Calibur's CPU time and obtained decoy scores using 31000 decoys

Target	TM-score	C_α RMSD	CPU Time
1abv_	0.2868	13.050	61.00
1af7__	0.4767	4.452	68.56
1ah9_	0.6450	3.314	1134.76
1aoy_	0.6649	4.724	2924.48
1b4bA	0.4728	5.751	118.88
1b72A	0.6764	3.102	80.77
1bm8_	0.3627	6.842	1363.30
1bq9A	0.3734	8.181	491.85
1cewI	0.1840	16.137	1191.57
1cqkA	0.8523	1.687	1530.71
1csp_	0.7190	2.384	277.44
1cy5A	0.8701	1.620	3343.29
1dcjA_	0.3701	12.182	485.11
1di2A_	0.7668	2.618	744.34
1dtjA_	0.7960	2.115	1399.34
1egxA	0.7740	2.598	1732.00
1fadA	0.5800	3.620	319.80
1fo5A	0.5432	3.871	1516.35
1g1cA	0.7794	2.648	2128.74
1gjxA	0.4104	7.830	85.20
1gnuA	0.5420	9.150	564.41
1gpt_	0.5113	6.292	1357.01
1gyvA	0.7678	3.435	294.76
1hbkA	0.6633	3.482	883.23
1itpA	0.3207	10.924	83.01
1jnuA	0.7506	2.681	1308.58
1kjs_	0.3830	8.436	501.81
1kviA	0.7067	2.100	970.08
1mla_2	0.6349	2.824	63.10
1mkyA3	0.4095	5.579	219.33
1mn8A	0.3480	7.452	133.68
1n0uA4	0.4571	4.623	231.90
1ne3A	0.5214	4.073	197.52
1no5A	0.4251	10.695	153.55
1npsA	0.7565	2.280	894.72
1o2fB_	0.3585	9.004	65.26
1of9A	0.5422	3.635	1289.87
1ogwA_	0.6844	2.717	1205.06
1orgA	0.7566	2.659	1636.53
1pgx_	0.5043	3.506	525.92
1r69_	0.7538	1.971	752.21
1sfp_	0.7455	5.261	2031.52
1shfA	0.8183	1.494	410.80
1sro_	0.6483	3.542	1165.09
1ten_	0.8233	1.837	1507.16
1tfi_	0.4983	5.077	1950.76
1thx_	0.7966	2.262	3726.24
1tif_	0.3084	7.871	76.55
1tig_	0.5609	3.580	95.28
1vcc_	0.3717	8.159	573.85
2a0b_	0.7745	2.782	1812.09
2cr7A	0.3699	8.239	3600.48
2f3nA	0.7263	1.938	68.14
2pcy_	0.6402	4.658	931.66
2reb_2	0.3372	5.928	1225.95
256bA	0.7600	3.448	77.62
	Avg. TM-score	Total C_α RMSD	Total CPU time
	0.581805	282.32	53582.2
